# Combined Efficacy of Balloon Occlusion and Uterine Artery Embolization on Coagulation Function in Patients with High-Risk Placenta Previa during Cesarean Section

**DOI:** 10.1155/2022/7750598

**Published:** 2022-04-04

**Authors:** Xiaoli Xu, Xiayun Zhu

**Affiliations:** Department of Obstetrics and Gynecology, Gezhouba Central Hospital of Sinopharm, China Three Gorges University, Yichang, Hubei, China

## Abstract

**Purpose:**

The present study was performed in order to investigate the conbined effect of balloon occlusion and uterine artery embolization on coagulation function in patients with high-risk placenta previa during cesarean section.

**Methods:**

There involved a total of 38 patients with high-risk placenta previa undergoing cesarean section in our hospital from August 2019 to January 2021. The patients enrolled were randomly divided into study group (19 cases, receiving balloon occlusion combined with uterine artery embolization) and control group (19 cases, receiving conventional cesarean section). The operation time, intraoperative blood loss, plasma injection volume and hospital stay of the two groups were recorded. Moreover, the postoperative coagulation function indexes, including thrombin time (TT), fibrinogen (FBI), activated partial thromboplastin time (APTT) and prothrombin time (PT), were monitored and compared. Neonatal Apgar score and postoperative complications of the two groups were regarded as parameters for comparison.

**Results:**

The intraoperative blood loss, plasma injection volume and hospital stay of the study group were significantly lower compared with the control group (*P* < 0.05), whereas the operation time of the two groups was comparable (*P* > 0.05). Compared with the control group, the levels of TT, APTT and PT were lower while the level of FBI was higher in the study group (*P* < 0.05). The Apgar 1-min and 5-min scores of newborns were compared between the two groups (*P* > 0.05). However, the incidence of postoperative complications in the study group showed evidently lower outcomes compared with the control group (*P* < 0.05).

**Conclusion:**

The combined approach of balloon occlusion and uterine artery embolization offered potential for improving the coagulation function of patients with high-risk placenta previa during cesarean section. In addition, the approach reduced the amount of blood loss and plasma injection, shortened the length of hospital stay, which was believed available for wide clinical application.

## 1. Introduction

With the publicly available information that we could find, the prevalence rate of placenta previa in foreign countries is about 0.3%–0.9%, and 0.2%–1.6% in China [1, 2]. As the number of maternal abortions has experienced a trend with gradual increase in recent years, the endometrial injury has increased the prevalence of placenta previa. Placenta previa is acknowledged as a complication of late pregnancy, which consists of pernicious and non-placenta accrete previa according to the degree of the disease. It can be divided into marginal, partial and central placenta previa based on the location of the lower edge of placenta and the internal orifice of the uterus. High-risk placenta previa includes placenta accrete previa and central placenta previa, which may increase the risk of aggravating the condition. Not only can it lead to intractable massive hemorrhage, but it can also damage surrounding organs, and even endanger the life of maternal and fetal [3, 4]. Therefore, additional focus is necessary for clinical practice on how to effectively treat patients with high-risk placenta previa.

Conventional cesarean section is currently used in treating patients harboring high-risk placenta previa. Methods for hemostasis include uterine tamponade, suture and oxytocin drugs, however, they fail to achieve ideal effects among some patients. Despite that several studies have showed the increasing use of balloon occlusion in the treatment of placenta previa in recent years, the method still can not completely stop the bleeding, and there were patients who suffered from active bleeding [5]. According to several studies, the combined application of balloon occlusion and uterine artery embolization can reduce the intraoperative and postoperative blood loss of patients with high-risk placenta previa undergoing cesarean section, as well as the risk of complications [6, 7]. However, limited researches are available in China. Herein, our study exerted efforts to investigate the overall effect of balloon occlusion combined with uterine artery embolization on coagulation function of patients with high-risk placenta previa during cesarean section, in order to provide insight for clinical practice in the management of the condition.

## 2. Materials and Methods

### 2.1. Clinical Background

In total, there included 38 patients with high-risk placenta previa undergoing cesarean section in our hospital from August 2019 to January 2021. The participants were randomly allocated into study group and control group, with 19 cases in each group. On one hand, in study group, the age ranged from 23 to 36 years old, with an average age of (28.83 ± 4.21) years old. The body mass index (BMI) was 23–28 kg/m^2^, and the average BMI was (25.23 ± 1.03) kg/m^2^. There were 2 to 5 times of pregnancy, with an average of (3.23 ± 0.76) times. There were 1 to 3 times of delivery, with average of (1.32 ± 0.19) times. The gestational age was 34 to 37 weeks, and the average of (36.87 ± 1.72) weeks. Classification: 11 cases of central placenta previa, 8 cases of placenta accrete previa. On the other hand, in the control group, the age ranged from 23 to 34 years old, with an average of (28.79 ± 4.19) years old. The body mass index was 23–27 kg/m^2^, and the average body mass index was (25.17 ± 1.01) kg/m^2^. There were 2 to 5 pregnancies with an average of (3.26 ± 0.74) times. There were 1 to 4 times of delivery, with an average of (1.31 ± 0.21) times. The gestational weeks ranged from 33 to 37 weeks, and an average of (36.79 ± 1.69) weeks. Classification: 10 cases of central placenta previa, 9 cases of placenta accrete previa. The general data of the two groups were comparable (*P* > 0.05). The formulation of this research protocol is in line with the relevant requirements of the Helsinki Declaration of the World Medical Association.

### 2.2. Selection Criteria

#### 2.2.1. Inclusion Criteria

(1) The patients were diagnosed with high-risk placenta previa for cesarean section based on the Obstetrics and Gynecology (9th Edition) [8]; patients in late pregnancy experienced painless vaginal bleeding with unknown causes, who were diagnosed with central placenta previa or placenta accrete previa using MRI [9] or B-ultrasound [10]. (2) Single live fetus. (3) There was no history of cesarean section in central placenta previa. (4) The coagulation function was normal. (5) The function of endocrine system was normal. (6) Complete clinical information. (7) All patients and their families had informed consent. (8) No allergy to contrast medium.

#### 2.2.2. Exclusion Criteria

(1) Patients with abnormal mental state and poor compliance. (2) Patients with severe malignant tumor. (3) Patients with abnormal circulatory system or liver and kidney function. (4) Patients with tuberculosis, syphilis, AIDS and other infectious diseases. (5) Previous thrombosis. (6) Patients with chronic pain or peripheral neuropathy. (7) The skin of abdominal wall was damaged and infected (8) Previous history of smoking, drinking, psychosis, motion sickness or postoperative nausea and vomiting. (9) Patients with other pregnancy complications.

## 3. Methods

The diagnosis and treatment mode of high-risk placenta previa were operated. Doctors from relevant departments, including neonatology department, imaging department, obstetrics department, vascular surgery department, urology department, interventional department, operating room, laboratory department, etc., should consult. Pain MRI and color Doppler ultrasound would be used to understand the placental location, implantation, scope and relationship with surrounding tissues, determine the location of uterine incision, and the appropriate operation time and operation plan. Enough blood was prepared. All parturients underwent elective surgery in operating room.

The control group was given conventional cesarean section. In operating room, patients were given general anesthesia. The placental attachment site was avoided as the uterine body incision to deliver the fetus. Routine disinfection was performed for cesarean section. After entering the abdomen, the uterine wall was cut, and the fetus was delivered and handed over to the neonatal physician for treatment. 10 U oxytocin was injected into the uterine body and maintained by intravenous injection of 10 U oxytocin. The placenta was stripped by hand. The placenta tissue on the placenta accreta surface was cleaned, the bladder peritoneum was separated, the bladder was pushed down, the lower uterine segment was exposed, the wound was sutured, and the myometrium was repaired. If uncontrollable bleeding occurs, the uterus will be removed. If there is no bleeding, conventional suture should be performed. In case of uterine atony, hemabate should be injected into uterine body in time. If excessive bleeding (more than 1000 ml) occurs continuesly, the anesthesiologists and ICU doctors should be responsible to maintain the vital signs of patients, giving anti fibrinolysis treatment, and giving warm liquid injection and keeping warm. The arterial blood gas (including K^+^, Ga^+^, hemoglobin, etc.) and central venous pressure should be monitored and recorded to maintain the blood pressure at 80/50 mmhg. The condition of patients was closely monitored to determine if they should transfer to the ICU or if blood transfusion was needed. After operation, the patients were given 2 U hemagglutinase.

The study group was given balloon occlusion combined with uterine artery embolization. The right femoral artery was punctured by the modified Seldinger method. The 16 mm balloon catheter and the 8 F artery sheath were inserted. 1–2 ml contrast medium was injected. The balloon was placed about 1 cm below the opening of bilateral renal artery. After the fetus was delivered and the umbilical cord was cut off, the balloon was filled. After the placenta was stripped and the placenta tissue was cleaned, the bleeding area of the uterine wall was sutured, and the contrast medium in the balloon was extracted. The balloon was filled every 5 minutes. After hemostasis, bilateral internal iliac arteriography was performed to determine the route of uterine artery. Selective catheterization of bilateral uterine artery was performed, and gelfoam particles were used for embolization. The bandage was removed after 24 hours in bed.

### 3.1. Observation Indexes

The operation time, intraoperative blood loss, plasma injection volume, and hospital stay were measured and recorded. The postoperative coagulation function indexes, including thrombin time (TT), fibrinogen (FBI), activated partial thromboplastin time (APTT) and prothrombin time (PT), were recorded. Neonatal Apgar score and postoperative complications were compared between the two groups.

Laboratory indicators: 3–5 ml elbow venous blood was collected from patients after operation, and stored in −30°C refrigerator after centrifugation. The levels of TT, FBI, APTT and PT were analyzed by automatic biochemical analyzer. The kits were purchased from Shanghai Hanfei Medical Instrument Co., Ltd. and strictly operated according to the reagent instructions.

Postoperative complications, including puerperal infection, poor wound healing, ICU transfer, lower limb thrombosis, disseminated intravascular coagulation and hysterectomy rate, were compared.

### 3.2. Statistical Methods

SPSS 21.0 software was utilized to analyze the data. The operation time, intraoperative blood loss, plasma injection volume, hospital stay, postoperative coagulation function and other measurement data were analyzed, expressed as $\bar{x}\pm s$ using *t* test. The counting data were expressed as percentage (%) and chi square *χ*^2^ was used. A *P* < 0.05 represents significant difference.

## 4. Results

Comparison of operation time, intraoperative blood loss, plasma injection volume and hospital stay between the two groups.

The intraoperative blood loss, plasma injection volume and hospital stay of the study group were significantly lower compared with the control group (*P* < 0.05). However, the operation times were comparable between the two groups (*P* > 0.05), as laid out Table 1.

### 4.1. Comparison of Coagulation Function between the Two Groups

The study group showed remarkably lower levels of TT, APTT and PT, and higher level of FBI than the control group (*P* < 0.05), as shown in Table 2.

### 4.2. Comparison of Apgar Score between the Two Groups

Apgar 1-min and 5-min scores of newborns were comparable between the two groups (*P* > 0.05), as shown in Table 3.

### 4.3. Comparison of Postoperative Complications between the Two Groups

Compared with the control group, the incidence of postoperative complications in the study group was significantly lower (*P* < 0.05), as laid out in Table 4.

## 5. Discussion

Placenta previa is an obstetric complication in which the placenta is inserted in the lower uterine segment, generally 28 weeks after pregnancy [11]. Recent studies have shown that, the incidence of high-risk placenta previa has experienced a climbing trend as the increase of cesarean section rate [12]. In addition, it was reported that the application of In Vitro Fertilization (IVF) also increased the risk of placental anomalies [13]. Severe postpartum hemorrhage is prone to occur in the process of cesarean section. Its main characteristics are intractable hemorrhage and placenta accreta. Improper treatment can pose a serious threat to the safety of mother and baby. A study [14] explicated that the median bleeding volume of patients with high-risk placenta previa ranged from 2000 ml to 7800 ml. In the past, hysterectomy was often used to treat intractable postpartum hemorrhage, but it failed to reduce the amount of bleeding, and even led to infertility. The operation of preserving uterus may result in massive hemorrhage and various complications. Hence appropriate management is of great significance to treat high-risk placenta previa, to reduce the amount of bleeding and the incidence of complications.

Based on a recent study [15], the application of interventional therapy in postpartum hemorrhage showed a good hemostatic effect. In 2009, the society of Obstetrics and Gynecology in abroad has encouraged obstetricians and interventional physicians to give interventional treatment to patients with high suspected or clear risk of massive hemorrhage of placenta accreta before surgery. Vascular interventional therapy is able to effectively stop bleeding by embolizing bleeding vessels directly. In recent years, as the advances of interventional therapy in placenta previa, balloon occlusion has been gradually acknowleged and applied in the treatment of placenta previa [16]. It was reported that preventive placement of balloon catheter was an effective method to manage severe hemorrhage of placenta previa, which could reduce intraoperative blood loss, perioperative hemostasis and reduce hysterectomy [17]. Due to the placental attachment site of patients with high-risk placenta previa, the physiological contraction function is reduced, and a large area of blood sinus is open, a large amount of bleeding may occur in a short time. Another study revealed that placenta previa was an independent risk factor for maternal bleeding [18]. Moreover, the blood can affect the operation due to unclear surgical field of vision. In addition, the placenta accreta muscle layer is difficult to peel off, which can lead to the aggravation of the disease. When the balloon is filled, it can block most of the pelvic blood supply below the abdominal aorta. Furthermore, it is conducive to the operation and reduce the operation time, in terms of slowing down the blood flow and reducing the intraoperative blood loss with clear surgical vision.

According to Qian et al. [19], the treatment of placenta accrete previa with abdominal aortic balloon occlusion combined with uterine artery embolization can effectively reduce the amount of blood transfusion and bleeding, shorten the operation time, the hysterectomy rate and the hospital stay. Wu et al. [20] pointed out, that the treatment of patients with placenta accrete previa by abdominal aorta balloon occlusion combined with uterine artery embolization can effectively reduce the amount of bleeding during operation, reduce the rate of hysterectomy and the incidence of postoperative complications. Based on our results, the amount of blood loss, plasma injection and hospital stay of the study group were markedly lower compared with the control group (*P* < 0.05), and the operation times of the two groups were comparable (*P* > 0.05). This suggested that balloon occlusion combined with uterine artery embolization is able to reduce the amount of blood loss and the amount of plasma injection, without increasing the operation time, which is also able to shorten the length of hospital stay. The results may be due to the rich blood supply of the uterus during pregnancy, mainly including uterine artery and other internal iliac artery branches, ovarian artery and other collateral circulation. Patients with high-risk placenta previa have abundant collateral circulation, abnormal proliferative blood vessels and dense venous plexus, hence uterine artery embolization alone can not achieve satisfactory hemostatic effect. It is necessary to block the blood flow of abdominal aorta embolization and its downstream branches with balloon occlusion before operation, so as to achieve satisfactory hemostatic effect and support the recovery of patients.

During pregnancy and delivery, the functions of coagulation, anticoagulation and fibrinolysis are changed among normal pregnant women. The levels of thrombin and coagulation factors in blood are increased, while the functions of anticoagulation and fibrinolysis are decreased, and the blood is in hypercoagulable state. The above functions are widely known as the natural protection function of pregnant women, which can effectively prevent postpartum hemorrhage and provide material basis for rapid hemostasis. Postpartum hemorrhage is a serious obstetric complication, especially in patients with high-risk placenta previa. The abnormal coagulation function of patients is an important factor leading to postpartum hemorrhage and disseminated intravascular coagulation [21]. Therefore, monitoring the coagulation function of patients with high-risk placenta previa can help effectively evaluate the risk of bleeding and hemostatic effect, which is pivotal for clinical practice. Our results demonstrated that the levels of TT, APTT and PT in the study group were lower, and the level of FBI was higher compared with the control group (*P* < 0.05), suggesting that balloon occlusion combined with uterine artery embolization can improve the coagulation function of patients with high-risk placenta previa during cesarean section, and prevent postpartum hemorrhage.

According to the results of our study, the Apgar 1-min and 5-min scores of newborns were comparable between the two groups (*P* > 0.05), suggesting that balloon occlusion combined with uterine artery embolization failed to affect the Apgar scores of newborns with high-risk placenta previa undergoing cesarean section. The reasons behind can be concluded as follows: (1) according to the International Commission on Radiation Protection (ICRP), the X-ray exposure dose of the fetus less than 100 mGy can not affect the tissue development function of the fetus, and the radiation dose in this study was less than the dose standard. (2) In addition, for patients with high-risk placenta previa during cesarean section, the balloon was not filled before the delivery of the fetus, hence the blood supply of the uterus was not blocked to affect the blood oxygen exchange between the uterus and placenta. Therefore, fetal intrauterine hypoxia can not occur, which may be an important reason for not affecting neonatal Apgar score.

Balloon occlusion is able to control the filling volume of balloon, and the operation is simple. It can effectively avoid the injury of vascular wall, prevent the formation of thrombosis and injury of arterial wall. After delivery of the fetus, the balloon is filled and the blood vessels below the abdominal aorta plane are blocked, which can effectively stop bleeding. After the hemostatic effect is achieved, the balloon is relaxed and the blood flow is restored, which can prevent the occurrence of complications such as lower limb ischemic necrosis caused by long-term compression [22]. Our results depicted that the incidence of postoperative complications in the study group was remarkably lower compared with the control group (*P* < 0.05), indicating that balloon occlusion combined with uterine artery embolization in the treatment of high-risk placenta previa patients with cesarean section had less postoperative complications and with safety profile. However, one patients suffered from lower limb thrombosis in the study group. Given that the patient was elderly maternal (36 years old), and discharged after thrombolytic therapy with longer postoperative bedridden time, it is worth noting that the blood was in a hypercoagulable state, and balloon occlusion may damage vascular intima. The condition and risk should be conprehensively evaluated before operation, including the skin color and dorsalis pedis artery pulse of patients after operation. And doctors should encourage patients to turn over early after operation, and carry out massage on lower limbs, to avoid the occurrence of related complications.

Taken together, the combined approach of balloon occlusion and uterine artery embolization elicited superior outcomes in the management of patients with high-risk placenta previa during cesarean section. Not only can it reduce the amount of blood loss and plasma injection, but it can also shorten the length of hospital stay, and improve coagulation function, without affecting neonatal Apgar score. In addition, the approach brought no risk for increasing the incidence of postoperative complications, which was believed available for clinical practice. However, considering the small sample size of this study, more studies are needed to further explore the effect of the balloon occlusion combined with uterine artery embolization on the fertility of patients.

## Figures and Tables

**Table 1:** Comparison of operation time, intraoperative blood loss, plasma injection volume and hospital stay between the two groups ($\bar{x}\pm s$).

Group	*n*	Operation time (min)	Intraoperative blood loss (ml)	Plasma injection volume (ml)	Hospital stay (d)

Control group	19	118.73 ± 18.73	803.28 ± 123.29	348.91 ± 37.98	7.03 ± 0.23
Study group	19	109.83 ± 17.27	1391.91 ± 132.87	763.29 ± 45.29	12.03 ± 0.34
*t*		1.523	−14.155	−30.559	−53.094
*P*		0.136	<0.001	<0.001	<0.001

**Table 2:** Comparison of coagulation function indexes between the two groups ($\bar{x}\pm s$).

Group	*n*	PT (s)	FBI (g/L)	APTT (s)	TT (s)

Control group	19	12.49 ± 1.08	3.12 ± 0.29	45.39 ± 2.76	19.02 ± 1.13
Study group	19	10.56 ± 1.02	3.99 ± 0.32	35.01 ± 2.33	15.13 ± 1.09
*t*		5.663	−8.781	12.526	10.799
*P*		<0.001	<0.001	<0.001	<0.001

**Table 3:** Comparison of Apgar score between the two groups ($\bar{x}\pm s$, points).

Group	*n*	1-min score	5-min score

Control group	19	8.87 ± 0.27	9.65 ± 0.23
Study group	19	8.91 ± 0.24	9.72 ± 0.15
*t*		−0.483	−1.111
*P*		0.632	0.274

**Table 4:** Comparison of postoperative complications between the two groups.

Group	*n*	Puerperal infection	Poor wound healing	ICU transfer	Lower limb thrombosis	DIC	Hysterectomy rate	Total incidence

Control group	19	0	1	3	0	0	3	7 (36.84)
Study group	19	0	0	0	1	0	0	1 (5.26)
*t*								5.699
*P*								0.017

## Data Availability

The datasets generated and analyzed during the current study are available from the corresponding author on reasonable request.
